# Editorial: Mitigating microbial contamination of drinking water sources

**DOI:** 10.3389/fmicb.2026.1779272

**Published:** 2026-02-16

**Authors:** Mohamed Azab El-Liethy, Sylvester Chibueze Izah, Godlisten N. Shao, Matthew Chidozie Ogwu, Ilunga Kamika

**Affiliations:** 1 Environmental Microbiology Laboratory, Water Pollution Research Department, National Research Centre, Giza, Egypt; 2 Department of Community Medicine, Faculty of Clinical Sciences, Bayelsa Medical University, Yenagoa, Bayelsa, Nigeria; 3 Department of Chemistry, Mkwawa University College of Education, University of Dar es Salaam, Iringa, Tanzania; 4 Goodnight Family Department of Sustainable Development, Appalachian State University, Living Learning Center, Boone, NC, United States; 5 College of Science, Engineering and Technology, Institute for Nanotechnology and Water Sustainability, University of South Africa, Johannesburg, South Africa

**Keywords:** aquatic environments, drinking water, microbial pathogens, mitigating microbial contamination, modeling study, water quality assessment

Human life depends primarily on water, yet there remains a shortage of clean water for domestic use, particularly among populations in low- and middle-income countries. An estimated 144 million people currently rely on untreated surface water, and over 785 million people globally lack access to safe drinking water (Bogale, 2020). Additionally, more than 2 billion people drink water contaminated by human waste and approximately 829,000 individuals, with children under the age of five being the most exposed, die from diarrhea each year as a result of poor hand hygiene, sanitation, and drinking water (Prüss-Ustün et al., 2019). The consequences include deadly waterborne infections, including cholera, dysentery, diarrhea, typhoid, and polio, which continue to affect vulnerable populations (Tsegaw et al., 2023). Common water-borne pathogens associated with illness include viral agents such as norovirus, rotavirus, astrovirus, and adenovirus; bacterial pathogens such as *Salmonella* spp., *Campylobacter* spp., pathogenic *Escherichia coli*, and *Shigella* spp.; and parasite infections such as *Cryptosporidium* and *Entamoeba* (Prasad and Grobelak, 2020).

Understanding the causes of fecal coliform contamination, such as poor sanitary practices and unsafe fecal sludge management, is crucial for designing effective water and sanitation safety plans to improve public health and nutrition in low and middle-income communities (Odey et al., 2017). To effectively mitigate microbial contamination of drinking water and enhancing water quality, a combination of strategies should be implemented. These include microbial water quality assessment and monitoring techniques, determination of sources and pathways of fecal contamination in water bodies, use of advanced microbial source tracking methods, finding low-cost technologies for detecting and mitigating microbial contamination, determining the health impacts of microbial contaminants in drinking water, designing predictive modeling of microbial contamination and associated health risks, and providing innovative wastewater treatment solutions for rural communities.

This Research Topic has 10 articles, including eight original research articles and two review articles, all of which were written by 54 authors from around the world, including Africa, Asia, Australia, Europe, and North America. Tang and Lau investigated the structure of the microbial community in the effluents of two sewage treatment works before and after the chlorination processes in Hong Kong. The researchers found that, the microbiomes show different resistance and resilience after chlorination steps. Additionally, highly dissimilar genomic and transcriptomic profiles based on their influent types (seawater or freshwater) have been shown. Furthermore, many genes linked to waterborne illnesses and antibiotic resistance were still present in the residual microbiomes in chlorinated effluents. In another study, Demirci et al. investigated the microbiomes in 52 water samples of which 18 samples were collected from drinking water treatment plants (DWTPs) and 34 collected from wastewater treatment plants (WWTPs) in Istanbul, Türkiye. The microbial metagenomic analysis revealed 71 phyla, 113 classes, 217 orders, 480 families, and 1,282 genera across all samples. Moreover, no antimicrobial resistance genes were detected in influlent and effluent of DWTPs. While, the resistance gene markers were detected in all effluent of WWTPs samples. Their findings revealed that there is no substantial microbiological risk difference between biological WWTPs and advanced biological WWTPs. Moreover, Wen et al. examined tap water microbiome profile and waterborne pathogens from different areas in China based on culture-independent 16S rRNA gene metabarcoding. Totally, 50 household drinking water samples were collected from 31 administrative regions spanning 19 provinces and regions in China. The results showed that, 44% of the total tap water samples was successfully amplified and with high DNA yield. A total of 7,635 microbial Amplicon Sequence Variants (ASVs) were discovered in 40 household drinking water samples from 27 cities in 19 provinces and regions of China. Their study emphasizes potential microbiological risks including potential bacterial pathogens and Microcystis in residential tap water, especially after extreme weather events. In a different study conducted by Hayward et al., the prevalence of opportunistic premise plumbing pathogens (OPPPs) was reported. These authors investigated the persistence of OPPPs using qPCR in drinking water plumbing systems, collecting 218 drinking water and 182 biofilm samples. They found that OPPs were detected in effcetive treated drinking water systems particullarly in biofilms. Also, there was positive correlation between the presence of *Acanthamoeba* and OPPPs. They also concluded that because variable water treatment and monitoring in residential settings may raise the risk of exposure to OPPPs, these findings are especially important for those receiving healthcare at home.

Khafaga et al. collected 30 well water samples from different locations in Sargodha City, Punjab Province, Pakistan. Physicochemical parameters were determined in the collected groundwater samples. In addition, the water quality index (WQI) was used to assess groundwater quality for drinking and irrigation. The findings showed that several groundwater samples had total dissolved solids (TDS), sodium (Na), potassium (K), and nitrate (NO_3_) concentrations exceeding the World Health Organization (WHO) acceptable limits. Furthermore, the average score of WQI was 84.57, which means that the quality of groundwater is poor and not suitable for drinking without a treatment step. The findings show that serious health issues, are influenced by groundwater pollution. The results emphasize the significance of focused water quality monitoring and public awareness campaigns to avert possible environmental and public health risks in those regions.

Nisar et al. tested various water disinfectants against clinically relevant free-living amoeba (FLA) that isolated from drinking water and biofilm samples. Thermal disinfection at 70 °C, hydrogen peroxide (H_2_O_2_), 2-methyl-4-isothiazolin-3-one, benzalkonium chloride (BAC), sodium hypochlorite, and chlorine dioxide (ClO_2_) were used as physical and chemical disinfectants with different disinfectant concentrations and contact times. The results showed that the thermal disinfectant was the most potent treatment followed by H_2_O_2_. Approximately >4 log_10_ of all FLA trophozoites were reduced by exposure to 70 °C for 45 min. *V. vermiformis* showed greater thermal tolerance than the other FLA.

Using Microbial Source Tracking (MST) and Illumina paired-end sequencing of bacterial 16S rRNA gene amplicons, Durstock et al. collected 24 samples to track fecal indicator bacteria (FIB) and possible waterborne pathogens in Sequiota Spring, Missouri, USA. Human fecal indicator bacteria (HFIB), especially *Bacteroides dorei*, were significantly reduced (46 times) between 2020 and 2022. Additionally, 16S rRNA gene sequences from the Enterobacteriaceae and Arcobacteraceae families also showed a reduction. Legionella-related sequences, however, did not change over the course of the investigation. The HFIB values in winter 2019 were equivalent to those in summer 2019, suggesting a similar pre-repair contamination. According to these findings, water quality was improved by fixing outdated sewer infrastructure, which significantly reduced human fecal contamination and the presence of waterborne pathogens.

On the other hand, the effectiveness of the solar photocatalyst of Bi TiO_2_-P25, designed by Arora et al. in lowering total coliforms from natural waters under solar light has been evaluated. Over 99% of total coliforms and *E. coli* were reduced within 2 h, according to the results. The reaction rate is greatly impacted by a number of variables, such as variations in the starting concentrations of pollutants in natural water and changes in sunlight intensity during treatment. The impact of these uncertainties on solar photocatalytic water treatment and treatment time data was investigated in their work study.

Dai et al. have written a systematic assessment of *Naegleria fowleri*'s biological traits and pathogenic mechanisms. Primary amoebic meningoencephalitis (PAM), a deadly infection of the central nervous system marked by quick clinical progression and a very high death rate, is caused by the uncommon pathogen *Naegleria fowleri*. They serve as a guide for the prevention and treatment of *N. fowleri* infections by offering theoretical support and useful advice for quick detection, precise diagnosis, and prompt intervention in clinical practice.

This Research Topic focuses on recent studies on water quality assessment, advanced microbial pathogen monitoring techniques, and surveillance and tracking of wastewater pollution and microbial contamination of domestic water resources, as well as microbial pathogen modeling solutions in aquatic environments. The significance of region-specific modeling techniques was highlighted by Izah and Ogwu. The effectiveness of predictive modeling methods in directing public health initiatives related to environmental matrices, such as prioritizing water purification efforts and putting early-warning systems in place during extreme weather events, was proved by case studies from South Asia and sub-Saharan Africa. Additionally, they investigate the incorporation of cutting-edge technologies, such artificial intelligence and remote sensing, into predictive frameworks, emphasizing their potential to enhance accuracy and scalability in environments with limited resources. It is advised to increase funding for data collection, predictive modeling tools, and cross-sectoral collaboration between governments, non-governmental organizations, and local communities. These initiatives are essential for creating robust water systems that can endure environmental stress and guarantee the long-term availability of clean drinking water. Stakeholders may efficiently handle microbiological contamination issues, protect public health, and help achieve the Sustainable Development Goals of the UN by utilizing predictive modeling as a fundamental element of water management plans.
